# Is Routine Monitoring of TNF-α Inhibitor Levels and Antibodies in Pediatric IBD Justified in the Era of Personalized Medicine

**DOI:** 10.3390/jcm15114098

**Published:** 2026-05-26

**Authors:** Tomasz Pytrus, Hubert Paweł Szyller, Gabriela Augustynowicz, Maria Lasocka, Sonia Watras, Katarzyna Akutko

**Affiliations:** 1 2nd Clinical Department of Paediatrics, Gastroenterology and Nutrition, Wroclaw Medical University, 50-369 Wroclaw, Poland; tomasz.pytrus@umw.edu.pl (T.P.); sonia.watras@umw.edu.pl (S.W.); katarzyna.akutko@umw.edu.pl (K.A.); 2 Student Scientific Group of Pediatric Gastroenterology and Nutrition, Wroclaw Medical University, 50-369 Wroclaw, Poland; gaba.augustynowicz12@gmail.com (G.A.); marysialasocka@gmail.com (M.L.)

**Keywords:** IBD, Crohn’s disease, ulcerative colitis, TDM, paediatric, infliximab, adalimumab

## Abstract

Inflammatory bowel disease (IBD) with an onset in childhood is characterized by a more extensive phenotype, a more aggressive clinical course, and a higher risk of long-term complications, including growth retardation, compared to adult-onset disease. While tumor necrosis factor-alpha (TNF-α) inhibitors are the cornerstone of therapy, achieving sustained remission in children is often hindered by unique pharmacokinetic challenges, such as accelerated drug clearance and a higher propensity for immunogenicity. This review explores the evolving role of therapeutic drug monitoring (TDM), specifically the paradigm shift from reactive to proactive strategies. While proactive TDM remains a subject of debate in adult IBD, emerging pediatric data strongly support its routine use to optimize treatment durability and prevent secondary loss of response. Evidence-based target trough concentrations for pediatric patients are critical for achieving mucosal healing: 8–13 µg/mL at week 6 and >5–7 µg/mL during maintenance for infliximab, and >13–14 µg/mL post-induction for adalimumab. Beyond clinical outcomes, this review emphasizes the economic viability of proactive TDM, which has been shown to reduce total healthcare expenditures by 18–30% by minimizing hospitalizations and avoiding premature treatment switches. By integrating pharmacological data with clinical pathways, proactive TDM serves as an essential tool for personalized medicine, ensuring safer and more cost-effective management of pediatric IBD.

## 1. Introduction

Inflammatory bowel disease (IBD), which includes Crohn’s disease (CD) and ulcerative colitis (UC), represents a growing challenge in the paediatric population. Epidemiological analyses from the last two decades indicate a steady increase in the incidence of IBD among children and adolescents, particularly in highly industrialised countries [1,2]. Paediatric patients account for over 20–25% of new IBD diagnoses, and in some countries the number of cases has doubled within a single generation [1].

IBD with childhood-onset is characterised by a more aggressive phenotype, greater inflammatory burden, faster progression, and more frequent involvement of a widespread section of the gastrointestinal tract compared with the adult form. Systemic consequences of chronic inflammation, such as growth disorders, delayed sexual maturation, and impaired bone mineralisation, are also more commonly observed in children, which represents one of the key therapeutic challenges and strongly justifies the need for rapid control of inflammatory activity [3,4].

Anti-tumour necrosis factor alpha (anti-TNF-α) drugs, including infliximab and adalimumab, form the cornerstone of treatment for moderate and severe IBD in children and have significantly improved rates of clinical and endoscopic remission [5,6]. In the IMAGINE 1 trial, adalimumab induction resulted in clinical response in 82.4% of paediatric patients with Crohn’s disease, while 27.7% achieved clinical remission after induction therapy. Clinical remission was maintained in 33.5% of patients at week 26 and in 28.4% at week 52 [5,6]. Despite proven efficacy, the response to treatment remains highly heterogeneous. A significant proportion of paediatric patients experience a primary lack of response or secondary loss of response within the first few years of therapy, which often necessitates dose escalation, switching to another biologic agent, or discontinuation of treatment. In the case of infliximab, approximately 10–30% of patients do not respond to induction therapy, and approximately 40–50% of initially responsive patients eventually lose their response [7,8,9]. Population-based long-term data demonstrate anti-TNF failure in approximately one-third of paediatric patients within the first year of therapy and in up to two-thirds within 5 years, with secondary loss of response representing the leading cause of treatment discontinuation [7].

Growing evidence suggests that this variability stems mainly from pharmacokinetic characteristics specific to the paediatric population and increased immunogenicity, leading to subtherapeutic drug exposure with standard dosing regimens, which are largely based on data from the adult population [10,11]. In children, faster drug clearance, dynamic changes in body composition, high inflammation intensity, and protein loss through the inflamed intestinal mucosa contribute to unstable drug exposure, particularly during the induction phase, where insufficient concentrations may critically determine the long-term durability of the therapeutic response [12,13,14]. Prospective paediatric studies further support the importance of adequate early infliximab exposure. In children with ulcerative colitis receiving infliximab induction therapy, clinical remission at week 8 was achieved in 70,6% of patients, while clinical response occurred in 82.4%. Importantly, higher infliximab concentrations during induction were associated with substantially improved rates of clinical response, mucosal healing, and clinical remission, highlighting the importance of early pharmacokinetic optimization [12].

Therapeutic drug monitoring (TDM) offers a pharmacokinetically guided approach to biologic therapy by integrating serum drug concentration measurements with the assessment of anti-drug antibodies [15]. Although reactive TDM is widely accepted as the standard tool for managing secondary loss of response, its preventive value is by definition limited, as it only allows the identification of treatment failure after clinical deterioration has occurred [16,17]. In contrast, proactive TDM aims to maintain therapeutic drug exposure before loss of response develops, which may be of particular importance in the paediatric population due to greater pharmacokinetic variability and a higher risk of immunogenicity [18,19].

In light of the above data, the question of the validity of routine monitoring of TNF-α inhibitor concentrations and specific antibodies in the paediatric population becomes of great clinical importance. A growing evidence base suggests that children—more than any other patient group—benefit from a precise, pharmacokinetic-based approach to treatment.

## 2. Clinical Applications of Therapeutic Drug Monitoring (TDM) in Pediatric Inflammatory Bowel Disease

The development of the concept of therapeutic drug monitoring (TDM) in TNF-α inhibitor therapy was initially based almost exclusively on data from adult populations [20]. These studies demonstrated a linear relationship between trough infliximab concentrations and clinical and endoscopic response, and unequivocally confirmed the significant impact of immunogenicity on secondary loss of treatment efficacy [21]. This formed the basis for the exposure-effect relationship concept in biologic therapy. However, the direct application of these observations to the pediatric population proved insufficient, due to differences in both pharmacokinetics and disease phenotype in children [20].

IBD with onset during childhood is more frequently characterized by a more aggressive course, greater inflammatory activity, and a higher risk of failure of biologic therapy compared to the adult population [3,4,22]. These phenomena, combined with the dynamic physiological changes of the growth period, influence the pharmacokinetic parameters of biologic drugs. Population studies have demonstrated greater variability in the clearance of infliximab and adalimumab in children [23,24,25]. A consequence of this variability is a significant risk of subtherapeutic exposure as early as the induction phase, particularly in patients with high inflammatory activity, hypoalbuminemia, and extensive gastrointestinal involvement [25,26]. In practice, this means that standard dosing regimens do not ensure uniform exposure in this population, which justifies the need for individualized therapy.

### 2.1. Exposure Response Relationship in the Pediatric Population

Cohort data in the pediatric population have shown a clear association between higher trough concentrations of infliximab during the induction phase and a greater likelihood of achieving long-term clinical remission and mucosal healing [9,19,27,28]. Particular prognostic significance is attributed to concentrations measured at weeks 6 and 14 of treatment. Values in the approximate range of 8–13 µg/mL at week 6 and >5–7 µg/mL at week 14 correlated with a more favorable clinical course and lower inflammatory activity during follow-up [28,29].

At the same time, in maintenance therapy, low trough concentrations of infliximab, most often <3 µg/mL, were more frequently observed in patients with persistent disease activity and the presence of anti-drug antibodies, indicating a close relationship between insufficient exposure, immunogenicity, and secondary loss of response [30,31].

Similar relationships have been demonstrated for adalimumab. Higher trough concentrations after completion of induction, exceeding 13–14 µg/mL, were associated with a higher likelihood of clinical remission and improvement in inflammatory markers, including CRP and calprotectin. In contrast, during maintenance therapy, concentrations < 5–7 µg/mL correlated with disease activity and an increased risk of anti-drug antibody production [32,33].

These data confirm that in the pediatric population, the clinical effect is strongly dependent on the level of exposure, and even short-term subtherapeutic exposure during the induction phase may negatively affect the long-term efficacy of biologic therapy. It should be emphasized that there are no clearly established, universal therapeutic thresholds for the pediatric population. The proposed ranges should be considered as guidelines to support the process of individualizing dosing within the framework of TDM, rather than as absolute values [12,28,29,32,33]. Although the exposure–response relationship for TNF-α inhibitors in pediatric IBD is consistently observed across studies, the predictive precision of individual trough concentration thresholds remains moderate and heterogeneous. In pediatric infliximab therapy, a week-14 trough concentration threshold of 6.9 µg/mL predicted sustained remission with a sensitivity of 58%, specificity of 81%, positive predictive value (PPV) of 86%, negative predictive value (NPV) of 50% with an area under the ROC curve (AUROC) 0.75, whereas a week-6 threshold of 13.2 µg/mL showed lower discriminatory performance—sensitivity 68%, specificity 69%, positive predictive value of 81%, negative predictive value of 52% and AUROC 0.67 [28]. Similar variability was observed in studies evaluating inflammatory biomarkers as indirect predictors of subtherapeutic exposure. An ESR ≥ 15 mm/h predicted undetectable infliximab trough concentrations with a sensitivity of 78%, specificity of 70%, PPV of 43%, and NPV of 90% [30]. In adalimumab-treated children, early drug exposure demonstrated strong predictive performance in selected cohorts. A week-4 adalimumab concentration threshold of 13.85 µg/mL predicted sustained remission at week 52 with a sensitivity of 83.3%, specificity of 88.8%, positive predictive value (PPV) of 83.3%, and negative predictive value (NPV) of 88.8%, with an AUROC of 88.9%. For prediction of sustained remission at week 82 using the same threshold, sensitivity was 100%, specificity 88.9%, PPV 83.3%, NPV 100%, and AUROC 95.6%. In addition, a week-22 adalimumab concentration threshold of 7.54 µg/mL predicted sustained remission at week 52 with a sensitivity of 83.3%, specificity of 80%, PPV 62.5%, NPV 92.3%, and AUROC of 78.9%, whereas a threshold of 10.51 µg/mL at week 22 predicted sustained remission at week 82 with a sensitivity of 57.14%, specificity of 91.66%, PPV 80%, NPV 78.57%, and AUROC of 73.8% [32].

A structured overview of the above data is provided in Table 1.

### 2.2. Immunogenicity and Prevention of Secondary Loss of Response

The immunogenicity of TNF-α inhibitors is one of the key mechanisms underlying the failure of biologic therapy in children [34]. In the pediatric population, there is a higher risk of anti-drug antibody (ADA) formation, earlier onset of ADAs, and a stronger association between the presence of ADAs and loss of clinical response [35]. The presence of ADAs leads to accelerated drug clearance, reduced trough concentrations, and limited efficacy of dose escalation [27].

Pediatric data indicate that maintaining stable, therapeutic concentrations of the TNF-α inhibitor significantly reduces the risk of immunogenicity. In this context, TDM serves not only as a reactive tool but also as a preventive one, reducing the risk of secondary loss of response by optimizing exposure [18,26].

### 2.3. The Importance of Early Exposure and the Induction Phase

A growing body of evidence supports the importance of early exposure to a TNF-α inhibitor for long-term treatment outcomes, in line with the “early exposure–long-term outcome” concept. Achieving adequate trough concentrations in the first weeks of therapy significantly reduces the risk of immunogenicity, loss of response, and the need for treatment intensification [28,32,36].

Particular prognostic significance is attributed to very early time points—during administration of the second and third infliximab induction doses. Children achieving clinical remission were characterized by significantly higher drug concentrations at these time points compared with non-responders [37,38]. Due to faster clearance and greater pharmacokinetic variability in the pediatric population, the importance of monitoring during induction is clearly greater than in the adult population, a fact reflected in current recommendations from scientific societies [12].

### 2.4. Is It Worthwhile to Routinely Monitor Concentrations of TNF-Alpha Inhibitors in the Paedatric Population?

Currently, there are two main strategies for the therapeutic monitoring of TNF-a inhibitors in IBD: reactive TDM (performed in the event of secondary loss of response) and proactive TDM (performed routinely to maintain optimal drug levels and prevent loss of response) [39,40].

The practical feasibility of implementing proactive TDM in routine clinical care is a subject of ongoing discussion. A significant factor is the availability of measurements; while centralized laboratory testing using ELISA remains the gold standard, it often involves a turnaround time of several days or even weeks [40,41]. This delay can be a major barrier to real-time clinical decision-making. Therefore, the clinical utility of TDM is closely linked to the accessibility of rapid testing methods. Point-of-Care Testing (POCT) and bench-top assays are emerging as reliable alternatives that eliminate the need to wait for results, potentially allowing for immediate dose adjustments during a single patient visit [41].

## 3. Mechanisms of Biological Activity and Pharmacokinetics of TNF-α Inhibitors in the Pediatric Population

Infliximab and adalimumab are IgG1 antibodies directed against TNF-α, a key pro-inflammatory cytokine that plays a central role in the pathogenesis of inflammatory bowel disease (IBD). Both drugs bind to both the soluble (sTNF-α) and membrane-bound (mTNF-α) forms of the cytokine, leading to the neutralization of its biological activity and the inhibition of its interaction with TNFR1 and TNFR2 receptors [42,43,44].

TNF-α blockade results in the inhibition of macrophage and monocyte activation, a reduction in the production of pro-inflammatory cytokines (including IL-1β, IL-6, IL-12, and IL-23), and a limitation of the differentiation and activity of Th1 and Th17 lymphocytes. Additionally, a reduction in the expression of adhesion molecules (ICAM-1, VCAM-1) in the endothelium and intestinal epithelium is observed, which limits the influx of inflammatory cells into the intestinal mucosa [43,45]. Consequently, there is a reduction in inflammatory infiltration, improvement in intestinal barrier integrity, and resolution of the inflammatory response at both the mucosal and transmural levels—which is of particular importance in Crohn’s disease.

An important additional mechanism of action of TNF-α inhibitors is the phenomenon known as reverse signaling. The binding of mTNF-α present on the surface of immune cells initiates intracellular signaling pathways leading to apoptosis of activated T lymphocytes and monocytes, as well as to the inhibition of pro-inflammatory cytokine production [42,44]. This mechanism is considered one of the key elements responsible for inducing deep remission, including mucosal healing.

Additionally, it has been demonstrated that anti-TNF therapy can modulate dendritic cell function and restore the balance between effector and regulatory responses within the intestinal immune system, which promotes long-term control of inflammation [43,44,45].

### 3.1. Early Exposure and the Induction Phase: Top-Down vs. Step-Up Approach

The debate between the “top-down” (TD) strategy initiating anti-TNF therapy early after diagnosis and the conventional “step-up” (SU) approach has been largely settled by high-quality pediatric data. The multicenter randomized controlled trial TISCH (Top-down Infliximab Study in Children with Crohn’s disease) provided landmark evidence: at week 10, clinical remission rates were significantly higher in the TD group (61%) compared to the SU group (39%, *p* = 0.033) [41]. More importantly, the TD approach led to vastly superior endoscopic remission rates (59% vs. 17%, *p* = 0.001), emphasizing that early biological intervention is critical for achieving deep mucosal healing before irreversible bowel damage occurs [41].

Long-term outcomes from the RISK study, a large prospective cohort, further support early intervention. Pediatric patients receiving anti-TNF therapy within 12 weeks of diagnosis were significantly less likely to develop penetrating complications (Hazard Ratio = 0.30, 95% CI 0.10–0.89) compared to those on immunomodulators or no early biologics [40]. These findings suggest that the induction phase is a “window of opportunity” where optimized drug exposure through TDM can fundamentally alter the disease trajectory in children.

### 3.2. Pharmacokinetics of TNF-α Inhibitors in the Pediatric Population

The pharmacokinetics of infliximab and adalimumab in the pediatric population are characterized by significant interindividual variability, which is considerably greater than that observed in adult patients with IBD [20,23,34,46]. Pharmacokinetic parameters, particularly clearance (CL) and volume of distribution (Vd), are modulated by demographic, clinical, and inflammatory factors, which directly influence the resulting pharmacological exposure.

The most important determinants include body weight, age, disease activity, albumin concentration, and the presence of anti-drug antibodies (ADA) [42,46]. In children, higher clearance of the TNF-α inhibitor is typically observed, leading to lower trough levels when using standard mg/kg-based dosing regimens. This phenomenon is particularly evident during the induction phase of treatment.

Hypoalbuminemia, a marker of severe inflammation and protein loss into the intestinal lumen, is one of the most significant factors increasing infliximab clearance [46,47]. Lower albumin concentrations are associated with an increased fraction of unbound drug, faster clearance of immune complexes, and increased leakage through the damaged intestinal barrier. Consequently, patients with severe, extensive IBD are particularly at risk of subtherapeutic drug concentrations as early as the first weeks of therapy.

Another significant factor in pediatric pharmacokinetic variability is dynamic organ development and the changing ratio of body weight to plasma volume, which affects the volume of distribution of monoclonal antibodies [20,48,49]. In younger children, standard doses may lead to a relatively greater dilution of the volume of distribution, resulting in lower peak and trough concentrations.

Population studies have shown that standard infliximab dosing (5 mg/kg) often fails to achieve target therapeutic concentrations during the induction phase in children with high inflammatory activity [12,20]. Consequently, current European guidelines allow for dose intensification in high-risk groups, emphasizing the need for individualized therapy from the very start of treatment [39,50]. This is summarized in Table 2.

### 3.3. The Effect of Inflammatory Phenotype and Immunogenicity on the Pharmacokinetic Variability of TNF-α Inhibitors

The heightened inflammatory process in pediatric IBD significantly increases the clearance of TNF-α inhibitors through disease-activity-dependent mechanisms. High TNF-α expression in the intestinal mucosa leads to the phenomenon of target-mediated drug disposition (TMDD), in which increased antigen availability results in enhanced drug binding and faster clearance of immune complexes (“TNF-sink effect”) [44,46,48].

Additionally, severe inflammation is associated with increased intestinal barrier permeability, protein loss, and enhanced immunoglobulin proteolysis, which contributes to reduced trough drug concentrations [46]. A correlation has been demonstrated between high CRP levels and hypoalbuminemia and increased infliximab clearance during the induction phase.

In children, where IBD often presents with high inflammatory activity at the time of diagnosis, these mechanisms may be particularly pronounced. This creates a negative feedback loop: insufficient exposure sustains inflammation, which in turn increases drug clearance. This phenomenon provides a strong rationale for early dose optimization and proactive TDM. Appropriate concentration values for the drug are listed in Table 3.

Immunogenicity also significantly influences the pharmacokinetic variability of TNF-α inhibitors. Immunogenicity is one of the main limitations to the efficacy of TNF-α inhibitor therapy in pediatric patients with IBD. The formation of anti-drug antibodies (ADA) leads to increased drug clearance, reduced trough concentrations, and secondary loss of clinical response [51].

Infliximab, as a chimeric antibody, exhibits greater immunogenic potential compared to the fully human adalimumab, although ADAs can develop with both drugs. In children, the incidence of immunogenicity may be higher than in adults, which is explained by greater immune system reactivity and a higher prevalence of a severe inflammatory phenotype at the start of therapy.

The development of ADAs may be transient or persistent. Neutralizing antibodies directly block the drug’s biological activity, whereas non-neutralizing antibodies accelerate its elimination by forming immune complexes. Both mechanisms result in reduced drug exposure and an increased risk of disease flare-ups.

The use of combination therapy with immunomodulators (e.g., thiopurines) reduces the risk of immunogenicity but is associated with a potential increase in the risk of adverse effects. In the pediatric setting, the decision to use combination therapy requires an individual assessment of benefits and risks.

The phenomenon of immunogenicity is one of the main justifications for implementing therapeutic drug monitoring (TDM), which enables early detection of ADAs and adjustment of treatment strategies [52].

### 3.4. The Importance of Therapeutic Exposure (Target Levels)

Optimal therapeutic exposure to TNF-α inhibitors is crucial for the effectiveness of treatment in pediatric patients with IBD. Higher trough levels of infliximab and adalimumab correlate with a higher likelihood of achieving clinical remission, mucosal healing, and sustained control of transmural inflammation [19,27,28,53].

Pediatric studies have shown that standard dosing regimens often fail to achieve target concentrations during the induction phase, particularly in children with a severe disease phenotype, hypoalbuminemia, or extensive bowel involvement. Suboptimal exposure promotes the development of ADA, which further reduces treatment efficacy [52,54].

The exposure-response relationship is well documented: children who achieve higher trough concentrations have a higher remission rate and a lower risk of secondary loss of response [12,19,27]. These data support the use of proactive TDM during the induction phase and maintenance monitoring in high-risk groups to ensure stable exposure and minimize immunogenicity.

### 3.5. Individualized Dosing, Therapeutic Drug Monitoring (TDM), and Clinical Recommendations in Pediatric IBD

The pediatric population with inflammatory bowel disease is characterized by greater pharmacokinetic variability and higher immunogenicity compared to adult patients [20,21,28,51,52,54]. High variability in drug clearance, the influence of inflammatory activity, hypoalbuminemia, and extensive intestinal involvement mean that standard dosing regimens for infliximab and adalimumab often fail to achieve target therapeutic concentrations, especially during the induction phase of treatment. Suboptimal trough concentrations promote the development of anti-drug antibodies (ADAs) and secondary loss of response [28,51,52,54].

Modern population pharmacokinetic (popPK) models and Bayesian forecasting enable the prediction of drug concentrations based on individual patient parameters, such as body weight, age, inflammatory activity, or albumin levels [9,55]. PopPK models constitute a quantitative framework for model-guided precision dosing and integrate individual clinical and pharmacokinetic variables to estimate drug clearance and predict future trough concentrations [56,57,58]. The Fasanmade popPK model demonstrated the best overall predictive performance, with a relative bias of −20.7% before TDM and 11.2% after TDM, and a reduction in relative root mean square error from 84.1% to 51.6% after incorporation of TDM, highlighting the added value of Bayesian updating in improving prediction accuracy. Bayesian forecasting combines prior population pharmacokinetic information with individual TDM results, enabling continuous refinement of pharmacokinetic estimates and individualized dose optimization during both induction and maintenance therapy [56,57,58].

Bevers et al. demonstrated high agreement between observed and model-predicted infliximab concentrations across multiple popPK models in children with IBD, with Bayesian forecasting significantly improving the prediction of subsequent trough concentrations. The Fasanmade model additionally showed strong discriminative performance for predicting infliximab trough concentrations ≥ 5 mg/L, with a sensitivity of 83.5%, specificity of 80%, and an AUROC of 0.870. Prediction of week-14 trough concentrations based on week-6 measurements was particularly accurate [56]. Furthermore, Bayesian dashboard systems further enhance model-guided precision dosing by integrating clinical, laboratory, and pharmacokinetic data to optimize infliximab therapy. In a study of 50 IBD patients, a prototype dashboard targeting trough concentrations of 3 μg/mL recommended dose or interval adjustments in 43/50 patients based on clinical data alone, compared with 44% under standard of care (5–10 mg/kg every 6–8 weeks). After inclusion of infliximab trough levels and anti-drug antibodies, adjustments were recommended in 48/50 patients (96%), while standard of care (SOC) dosing was appropriate in only 22%. These findings demonstrate that Bayesian-guided dashboards more frequently support individualized dosing than conventional regimens [40,41]. These systems continuously refine pharmacokinetic parameters as new TDM data become available, improving predictive precision over time. In pediatric patients, this adaptive feature is particularly relevant due to dynamic changes in weight, albumin levels, and disease activity influencing drug disposition [40,41,59].

These models allow for dynamic dose adjustment, faster achievement of target levels, stabilization of drug exposure, and reduction of the risk of immunogenicity. In clinical practice, the implementation of these tools enables precise individualization of therapy, especially in the pediatric population with a severe disease phenotype.

Therapeutic drug monitoring (TDM) is a fundamental tool in pediatrics. The proactive strategy involves monitoring during the induction phase and throughout maintenance therapy in high-risk groups, which helps prevent subtherapeutic exposure and the development of ADA. A reactive strategy is used in cases of loss of clinical response and allows for differentiation between insufficient exposure and immunogenicity [13,28,51,52,54,60].

Population-based studies and real-world evidence show that proactive TDM leads to:

- reduced immunogenicity,
- fewer dose escalations,
- fewer hospitalizations,
- more stable drug concentrations [9,60].

Clinical recommendations state:

- ESPGHAN/ECCO (2020)—children exhibit greater pharmacokinetic variability and rapid clearance; a proactive strategy is recommended in cases of high risk of immunogenicity [55].
- NASPGHAN (2025)—proactive TDM during the induction phase is highly recommended; monitoring during the maintenance phase is recommended in children with rapid clearance or low trough concentrations [13].
- AGA (2017)—primarily reactive TDM; a proactive strategy is acceptable in selected cases; target concentration values adapted for pediatrics [55].

Economic analyses indicate that proactive TDM reduces total treatment costs by 18–30%, primarily by limiting unjustified changes in biologic therapy, hospitalizations, and surgical interventions [9,61]. The absence of TDM contributes to premature treatment escalation, increased immunogenicity, and higher healthcare costs [61].

### 3.6. Practical Feasibility and the Role of Point-of-Care Testing (POCT)

The widespread implementation of proactive TDM is often limited by the logistical constraints of traditional laboratory methods. Enzyme-linked immunosorbent assay (ELISA), the current gold standard, typically requires specialized equipment and batching of samples to be cost-effective, resulting in a turnaround time of several days or even weeks. This delay necessitates a second clinical encounter or a remote consultation to adjust the medication dose, which significantly reduces the efficiency of a personalized treatment strategy.

Rapid Point-of-Care Testing (POCT) assays, including lateral flow and bench-top technologies, offer a solution by providing results within 15–30 min. Recent studies and position papers, including the latest 2025 NASPGHAN statement [41], indicate that POCT results for both infliximab and adalimumab concentrations show high correlation and excellent agreement with traditional ELISA methods. The reliability of these rapid tests allows for a “test-and-treat” approach, where dose adjustments are performed during the infusion visit based on real-time drug levels. While POCT may have higher per-test costs compared to batch ELISA, the reduction in clinical workload and the potential for superior disease control through immediate optimization make it a feasible and highly attractive tool in pediatric gastroenterology.

### 3.7. The Challenge of Non-Universal Therapeutic Thresholds

A major point of contention in the clinical application of TDM is the lack of universally accepted therapeutic thresholds for drug concentrations. Critics of routine monitoring often point to the significant overlap in drug levels between responders and non-responders as one of the most substantial arguments against the systematic use of TDM. However, this variability deserves deeper discussion, as it stems from complex physiological and clinical factors rather than a failure of the method itself [39,41].

Firstly, therapeutic targets are not static and depend heavily on the desired clinical endpoint. For instance, achieving clinical remission typically requires lower drug concentrations than those needed for complete mucosal healing or the resolution of complex perianal fistulas. Secondly, the inflammatory burden varies significantly between patients; a child with severe, extensive disease and low albumin levels will have higher drug clearance, necessitating a higher concentration to achieve the same biological effect compared to a patient with milder disease [41]. Furthermore, assay variability between different manufacturers (e.g., traditional ELISA vs. rapid POCT) can lead to discrepancies in reported values, complicating the establishment of a single, universal “cut-off” point.

Rather than viewing the lack of universal thresholds as a limitation, it should be interpreted as a requirement for further treatment personalization. Current consensus guidelines, most notably the 2025 NASPGHAN position paper [41], have shifted the focus from fixed thresholds to individualized target ranges (e.g., 5–10 µg/mL for infliximab maintenance). This approach allows clinicians to adjust the target based on the specific patient’s disease activity, biomarker levels, and long-term therapeutic goals, effectively addressing the “one-size-fits-all” criticism often leveled against TDM.

The recommendations of the associations are shown in Table 4.

### 3.8. Phenotype-Specific TDM and Economic Considerations

The clinical utility of TDM is increasingly recognized in managing specific, high-risk IBD phenotypes. In pediatric patients with perianal Crohn’s disease, standard trough levels may be insufficient; emerging evidence suggests that higher infliximab concentrations (e.g., >10 µg/mL) are associated with superior fistula healing rates compared to those required for luminal remission alone [41]. Similarly, in cases of acute severe ulcerative colitis (ASUC), where drug clearance is significantly accelerated due to high inflammatory burden and fecal loss of the drug, intensified TDM-guided induction is crucial to prevent early colectomy. Tailoring TDM strategies to these specific phenotypes represents the next step in the evolution of personalized pediatric care.

From an economic perspective, while the initial costs of routine drug and antibody testing may seem high, proactive TDM has been shown to be highly cost-effective. Economic evaluations indicate that proactive monitoring reduces long-term healthcare expenditures by decreasing the frequency of flares, hospitalizations, and the need for expensive surgical interventions. Furthermore, TDM allows for the identification of patients with supratherapeutic drug levels (SSTL), enabling safe dose de-escalation. This “optimization downwards” significantly reduces the cost of expensive biologics without compromising clinical stability. By preventing secondary loss of response and extending the “lifespan” of the first-line anti-TNF agent, proactive TDM avoids the higher costs associated with switching to more expensive, second-line biological therapies [39,40,41].

## 4. Conclusions

Data from the literature clearly indicate that children with inflammatory bowel disease (IBD) constitute a population with a unique pharmacokinetic and immunological profile [20,28,51,52,54]. The high variability in TNF-α inhibitor clearance, associated with body weight, age, inflammatory activity, hypoalbuminemia, and extensive intestinal involvement, means that standard dosing regimens, based on data from the adult population, often lead to subtherapeutic drug concentrations during the induction phase. Such concentrations increase the risk of anti-drug antibody (ADA) formation and secondary loss of clinical response [19,34,51,52,54].

The exposure-response relationship is well documented in pediatrics. Higher trough concentrations of anti-TNF drugs correlate with a greater likelihood of clinical remission, mucosal healing, and long-term treatment stability [27,34,51]. The randomized PAILOT trial demonstrated the superiority of a proactive adalimumab TDM strategy over a reactive approach in terms of maintaining remission and reducing immunogenicity [51]. These results were confirmed in real-world studies, which demonstrated, among other things, a reduction in the number of hospitalizations, a decrease in dose escalation, and stabilization of drug concentrations [9,60].

An analysis of economic data clearly shows that proactive TDM is cost-effective, leading to an 18–30% reduction in total costs in the pediatric population [9]. The lack of drug concentration monitoring contributes to premature dose escalation, increased immunogenicity, and higher treatment costs [61]. These data underscore that TDM in pediatrics has both clinical and economic significance.

Conclusions from clinical and economic analyses unequivocally support the routine use of a proactive strategy, particularly in children with a severe disease phenotype, high drug clearance, or low minimum concentrations [13,55,60]. A reactive strategy remains essential in the event of loss of clinical response, allowing for differentiation between suboptimal exposure and immunogenicity [13,55,60].

Limitations of the available studies include primarily population heterogeneity, methodological differences in drug concentration measurement, and a limited number of randomized trials in children. Future directions include further integration of Bayesian forecasting models into routine clinical practice, standardization of therapeutic thresholds, development of rapid and cost-effective tests, and expansion of research on the efficacy of TDM in various IBD phenotypes in pediatrics [9].

In summary, the discussion indicates that the individualization of TNF-α inhibitor therapy using proactive TDM is crucial for:

- maintaining adequate drug exposure,
- reducing immunogenicity,
- improving clinical outcomes,
- optimizing treatment costs in the pediatric IBD population.

## Figures and Tables

**Table 1:** Predictive value of TNF-α inhibitors trough levels for sustained remission in pediatric IBD [28,32].

TNF-α Inhibitor	Week	Cut-Off (µg/mL)	Endpoint	Sensitivity	Specificity	PPV	NPV	AUROC
Infliximab	6	13.2	sustained remission	68%	69%	81%	52%	0.67
Infliximab	14	6.9	sustained remission	58%	81%	86%	50%	0.75
Adalimumab	4	13.85	week 52 remission	83.3%	88.8%	83.3%	88.8%	0.889
Adalimumab	4	13.85	week 82 remission	100%	88.9%	83.3%	100%	0.956
Adalimumab	22	7.54	week 52 remission	83.3%	80%	62.5%	92.3%	0.789
Adalimumab	22	10.51	week 82 remission	57.14%	91.66%	80%	78.57%	0.738

**Table 2:** The table presents the main factors influencing the pharmacokinetics of biologic drugs, with particular emphasis on changes in clearance (CL) and volume of distribution (Vd). The most important determinants include body weight, patient age, severity of inflammation, albumin concentration, and the presence of anti-drug antibodies (ADA). These factors can significantly alter drug exposure, affecting its clinical efficacy and the risk of treatment failure.

Determinant	Effect on Pharmacokinetics	Mechanism
Body weight	Higher body weight -> larger Vd, lower concentrations	Drug dilution in a larger volume of distribution
Age	Younger children -> higher clearance	Developmental physiological differences, including higher weight-normalized clearance
Inflammatory activity	High inflammatory activity -> increased clearance	Target-mediated drug disposition (TNF sink), intestinal leakage
Albumin concentration	Low albumin concentration -> higher clearance	Reduced drug-protein binding and faster elimination
Anti-drug antibodies (ADA)	Presence of ADA -> increased clearance	Drug neutralization and accelerated elimination

**Table 3:** Recommended target trough concentrations for Infliximab and Adalimumab. Adapted from Refs. [39,40,41] under CC BY 4.0 license.

Drug	Treatment Phase	Target Trough Concentration (µg/mL)	Clinical Goal
Infliximab	Induction (Week 2)	>20–25	Early response and long-term durability
	Induction (Week 6)	>15	Predicting clinical and endoscopic remission
	Maintenance	5–10	Clinical remission
	Maintenance (High Burden)	>10	Mucosal healing and fistula closure
Adalimumab	Induction (Week 4)	>5–10	Clinical response at Week 12
	Maintenance	5–8	Clinical remission
	Maintenance (High Burden)	>8–12	Deep remission and mucosal healing

**Table 4:** Comparison of TDM recommendations from major international guidelines.

Organization	Recommendation Type	Preferred TDM Strategy	IFX Maintenance Target (µg/mL)
ESPGHAN/ECCO	Pediatric-specific	Proactive TDM (especially during induction)	>5
NASPGHAN (2025) [41]	Pediatric-specific	Proactive TDM as standard of care	5–10
AGA	General (Adult-based)	Reactive TDM (conditional proactive)	≥5
British Society of Gastroenterology	General	Proactive TDM (post-induction)	3–7

## Data Availability

No new data was created.
